# Enhanced Visible-Light Photocatalytic Performance of SAPO-5-Based g-C_3_N_4_ Composite for Rhodamine B (RhB) Degradation

**DOI:** 10.3390/ma12233948

**Published:** 2019-11-28

**Authors:** Lingfang Qiu, Zhiwei Zhou, Mengfan Ma, Ping Li, Jinyong Lu, Yingying Hou, Xiangshu Chen, Shuwang Duo

**Affiliations:** 1 Jiangxi Key Laboratory of Surface Engineering, Jiangxi Science and Technology Normal University, Nanchang 330013, China; qlf1108@163.com (L.Q.); pharmacy_zhou@163.com (Z.Z.); MF1783975@126.com (M.M.); lp1849065552@163.com (P.L.); ljy1873642671@163.com (J.L.); hyy18170401838@163.com (Y.H.); 2 Institute of Advanced Materials (IAM), State-Province Joint Engineering Laboratory of Zeolite Membrane Materials, College of Chemistry and Chemical Engineering, Jiangxi Normal University, Nanchang, 330022, China; cxs66cn@jxnu.edu.cn

**Keywords:** g-C_3_N_4_, SAPO-5, composite, photocatalytic, visible light

## Abstract

Novel visible-light responded aluminosilicophosphate-5 (SAPO-5)/g-C_3_N_4_ composite has been easily constructed by thermal polymerization for the mixture of SAPO-5, NH_4_Cl, and dicyandiamide. The photocatalytic activity of SAPO-5/g-C_3_N_4_ is evaluated by degrading RhB (30 mg/L) under visible light illumination (λ > 420 nm). The effects of SAPO-5 incorporation proportion and initial RhB concentration on the photocatalytic performance have been discussed in detail. The optimized SAPO-5/g-C_3_N_4_ composite shows promising degradation efficiency which is 40.6% higher than that of pure g-C_3_N_4_. The degradation rate improves from 0.007 min^−1^ to 0.022 min^−1^, which is a comparable photocatalytic performance compared with other g-C_3_N_4_-based heterojunctions for dye degradation. The migration of photo-induced electrons from g-C_3_N_4_ to the Al site of SAPO-5 should promote the photo-induced electron-hole pairs separation rate of g-C_3_N_4_ efficiently. Furthermore, the redox reactions for RhB degradation occur on the photo-induced holes in the g-C_3_N_4_ and Al sites in SAPO-5, respectively. This achievement not only improves the photocatalytic activity of g-C_3_N_4_ efficiently, but also broadens the application of SAPOs in the photocatalytic field.

## 1. Introduction

For the society’s development and rapid industrialization, environmental pollution becomes much more serious. Noticeably, water purification requiring advanced technologies draws much attention in the research community. Photocatalysis is regarded as a superior approach for water treatment because of its unique advantages, such as non-secondary pollution, simple operation, short reaction period, and mild environment.

Graphitic carbon nitride (g-C_3_N_4_), a novel polymeric semiconductor, has become a new generation in the photocatalytic arena. Meaningful, characters like visible-light (over 43% of the solar energy) response, low cost, and ultrahigh physicochemical stability are believed to make g-C_3_N_4_ attractive for researchers. However, the mortal shortage, namely the narrow band gap energy (2.7 eV) leading to quick photo-induced electron-hole pair recombination, hinders the practical application of g-C_3_N_4_ [1]. Thus, g-C_3_N_4_-based heterojunction with resembled band structure promotes many urgent questions. Many semiconductors with a wider band gap have been applied in constructing g-C_3_N_4_-based heterojunctions, such as traditional TiO_2_ [2], ZnO [3], CdS [4], and so on.

Zeolites or zeolitic materials, a big family of porous materials, show many distinct properties, such as framework component controllability and high adsorption. Aluminosilicalites and aluminosilicophosphates (SAPOs) are usually used as catalysts in cracking, isomerization, and alkylation reactions, such as SAPO-34 [5,6], Y-type [7,8], and ZSM-5 [9]. Recently, it was found that aluminosilicalites and SAPOs also exhibited photocatalytic activity for the charge transfer excited state of [Al^2+^–O^−^]* under UV illumination, which has been demonstrated in some literature [9,10]. SAPO-5 has successfully been used as a direct photocatalyst for CO_2_ energy conversion and organic pollutant degradation owing to the special electron transfer pattern within activated [Al^2+^–O^−^]* sites where Al behaves as the electron acceptor and O acts as the electron donor. Furthermore, SAPO-5 has been applied in heterojunction-like construction with TiO_2_ [11], but the photocatalytic performance still needs to be improved and the photocatalytic mechanism is still not very clear.

In our previous study, SAPO-5 with a high ratio of Si/Al and nanometer-sized particles (high-silica SAPO-5) was found to have good photocatalytic activity under UV illumination for vast active sites [12]. Herein, we propose to construct a novel heterojunction-like composite by combining as-synthesized high-silica SAPO-5 with g-C_3_N_4_ nanosheet. Photodegradation for rhodamine B (RhB) will be chosen as the model to evaluate the photocatalytic activity of SAPO-5/g-C_3_N_4_. As far as we know, it is the first time modifying g-C_3_N_4_ with SAPO-5. The effects of SAPO-5 incorporation proportion and initial RhB solution concentration on the physicochemical properties and optical properties of SAPO-5/g-C_3_N_4_ will be analyzed by a series of characterizations and photodegradation pattern will be discussed in detail. Finally, the RhB degradation mechanism of SAPO-5/g-C_3_N_4_ will be proposed.

## 2. Experimental

### 2.1. Materials

Ammonium chloride (Damao Chemical Reagent Factory, Tianjin, China), dicyandiamide (Sinopharm Chemical Reagent Co., Ltd., Shanghai, China), ultra-pure water (made in the lab), and rhodamine B (Aladdin, Shanghai, China) were used in the following experiments.

### 2.2. Synthesis of SAPO-5/g-C_3_N_4_ Composite

SAPO-5/g-C_3_N_4_ composites were prepared simply by co-thermal polymerization. High-silica SAPO-5 has already been made in our laboratory and we use as-synthesized high-silica SAPO-5 directly in the subsequent experiments. Pure g-C_3_N_4_ nanosheet marked as CN-0 was synthesized according to the literature [13]. Typically, 15 g of NH_4_Cl and 3 g of dicyandiamide were dissolved in 50 mL ultra-pure water and then the mixture was evaporated at 80 °C under stirring until dry white solids formed again. Later, the mixture was transferred to a crucible with a cover and calcined at 500 °C for 4 h in a muffler. Either the heating rate or the cooling rate is 2 °C/min. To fabricate SAPO-5/g-C_3_N_4_ composite, SAPO-5 crystals were added into a mixture containing water, NH_4_Cl, and dicyandiamide, as described above. The mass ratios of SAPO-5 and the precursor of g-C_3_N_4_ were adjusted from 0% to 0.11%, 0.44%, 1.67%, 2.78%, 4.44%, and 5.56%, respectively. It should be noted that other treatments compared with pure g-C_3_N_4_ preparation were not changed. The final SAPO-5/g-C_3_N_4_ composites with different component contents (Table S1) were marked as SAPO-5/g-C_3_N_4_-X (X = 1, 2, 3, 4, 5, 6), successively.

### 2.3. Characterization

X-ray diffraction (XRD) (Shimadzu XRD-6100, Kyoto, Japan) was used for confirming the crystal phase of samples. A scanning electron microscope (SEM) (Zeiss Sigma, Jena, Germany) was applied to investigating the microstructure of samples and the contact surface between the SAPO-5 and g-C_3_N_4_ nanosheets in low resolution. The transmission electron microscope (TEM) (Tecnai G220 S-TWIN, Hillsboro, RO, USA) provides microstructure images with a much higher resolution of the g-C_3_N_4_ nanosheet and the SAPO-5/g-C_3_N_4_ hybrid structure. Surface groups of samples were detected by Fourier transform infrared spectroscopy (FT-IR) (Spectrometer PerkinElmer Spectrum Two, Waltham, MA, USA). UV-visible diffuse reflection spectra (DRS) (EscalLab 250 Xi, Shanghai, China), photoluminescence (PL) (Zolix LSP-X500A, Beijing, China), photocurrent (CHI660E, Shanghai, China), X-ray photoelectron spectroscopy (XPS) (ThermoFisher K-Alpha, Shanghai, China), and time-resolved fluorescence spectra (C11367, Quantaurus-Tau, Hamamatsu, Japan) were applied for analyzing a series of optical properties of the photocatalysts, which will be discussed below in detail.

### 2.4. Photocatalytic Experiment

Visible-light photocatalytic experiments were carried out within an optical reaction apparatus (XPA Xujiang, Nanjing, China) using a xenon lamp with a power of 500 W to produce visible radiation. The photocatalysts were applied to a degrading RhB aqueous solution. The physical adsorption equilibrium was achieved after 1 h in a dark environment. After that, the xenon lamp was turned on and samples were extracted every 20 min. The absorbance of dye solution after centrifugation was marked as A, which is proportional to dye concentration C. The initial absorbance and dye concentration were A_0_ and C_0_, respectively.

## 3. Results and Discussion

### 3.1. Characterization of Samples

**Figure 1:**
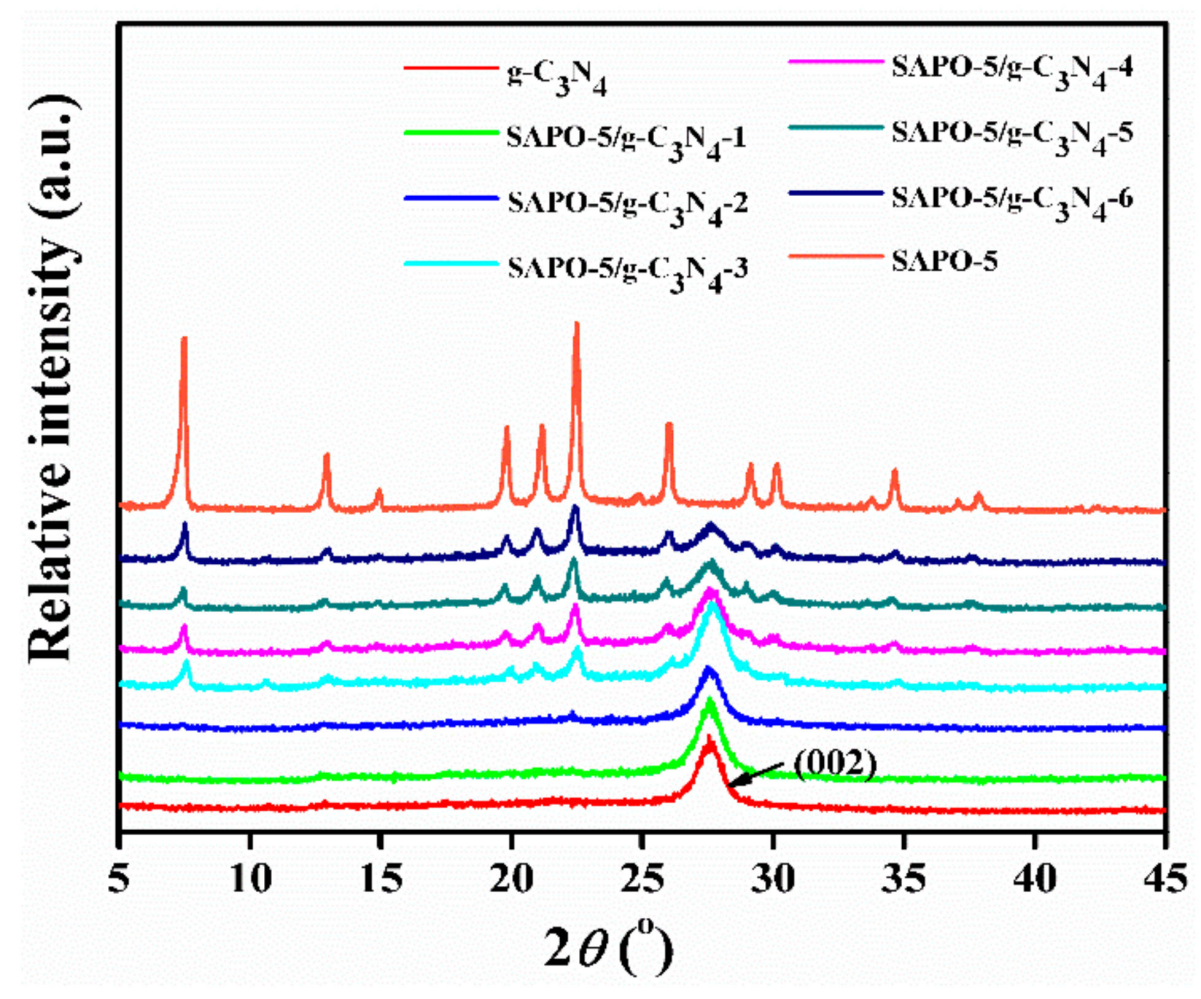
XRD patterns of pure g-C_3_N_4_ and aluminosilicophosphate-5 (SAPO-5)/g-C_3_N_4_ composites with different SAPO-5 incorporation proportions and pure SAPO-5.

Figure 1 showed the XRD patterns of pure g-C_3_N_4_, SAPO-5, and SAPO-5/g-C_3_N_4_ composites with different SAPO-5 incorporation proportions. The pattern for g-C_3_N_4_ indicated that pure g-C_3_N_4_ was prepared successfully, and the main peak located at 2*θ* = 27.7^o^ represented the (002) plane of g-C_3_N_4_, which agreed well with the data reported [14]. In addition, the interlayer stacking of synthesized g-C_3_N_4_ was 0.322 nm, which was similar to previously reported g-C_3_N_4_ [13]. All peaks in the XRD pattern of SAPO-5 were totally in tune with the typical peaks of pure SAPO-5 in reports [10,15]. After composites of SAPO-5/g-C_3_N_4_ were formed, the XRD patterns became the combination of pure g-C_3_N_4_ and SAPO-5. According to the corresponding patterns, we found that when the incorporation proportion of SAPO-5 was too small, it was difficult to detect the SAPO-5 crystal phase. As the incorporation proportion increased, the intensity of g-C_3_N_4_ was covered by SAPO-5, generally. Moreover, there was no impurity formed during the synthesis process of the composite photocatalysts, and the crystallinity of SAPO-5 was maintained perfectly.

**Figure 2:**
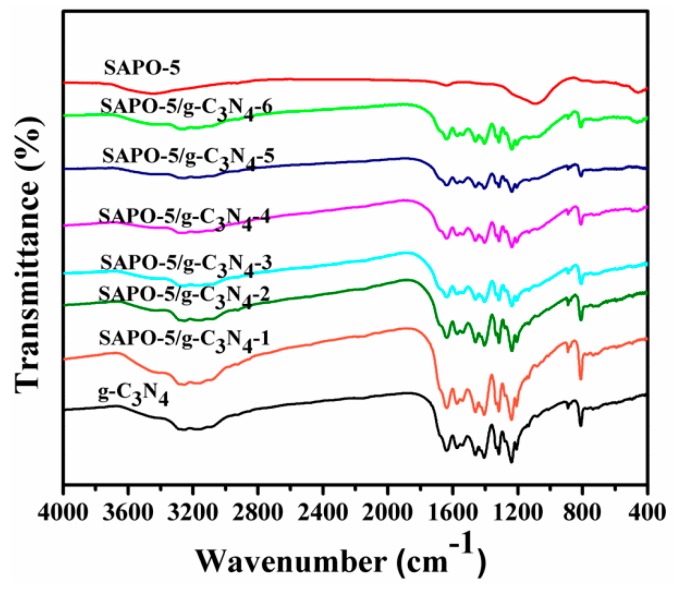
FT-IR patterns of pure g-C_3_N_4_ and SAPO-5/g-C_3_N_4_ composites with different SAPO-5 incorporation proportions and pure SAPO-5.

FT-IR spectra were applied for confirming the components of synthesized samples. As shown by the spectrum of pure g-C_3_N_4_ in Figure 2, the obvious peak at 810 cm^−1^ was from the stretching vibration of tri-s-triazine rings [16,17]. The strong peaks located within the range 1650–1200 cm^−1^ originated from the stretching vibration of aromatic C–N heterocycles [18,19]. The broad peaks representing the stretching vibration of NH groups were at 3000–3400 cm^−1^ [20]. Based on these distinctive peaks, it was demonstrated that g-C_3_N_4_ was indeed formed. Moreover, it should be noticed that the peak at 1640 cm^−1^ came from the unavoidable physical adsorption of O–H [21]. For pure SAPO-5, the corresponding spectrum indicated that peaks at 475 cm^−1^, 1115 cm^−1^, and 3486 cm^−1^ were assigned to the benching vibration of primary structural units (like SiO_4_ and AlO_4_), the stretching vibration of Si–O–Si, and the presence of P–OH, respectively [22,23]. It was obvious that during the formation of the SAPO-5/g-C_3_N_4_ composites, the component of g-C_3_N_4_ was well maintained. Furthermore, the peaks of SAPO-5 appeared as the incorporation proportion increased, which was well in accordance with Figure 1. It can be determined that SAPO-5 has been closely integrated with g-C_3_N_4_.

**Figure 3:**
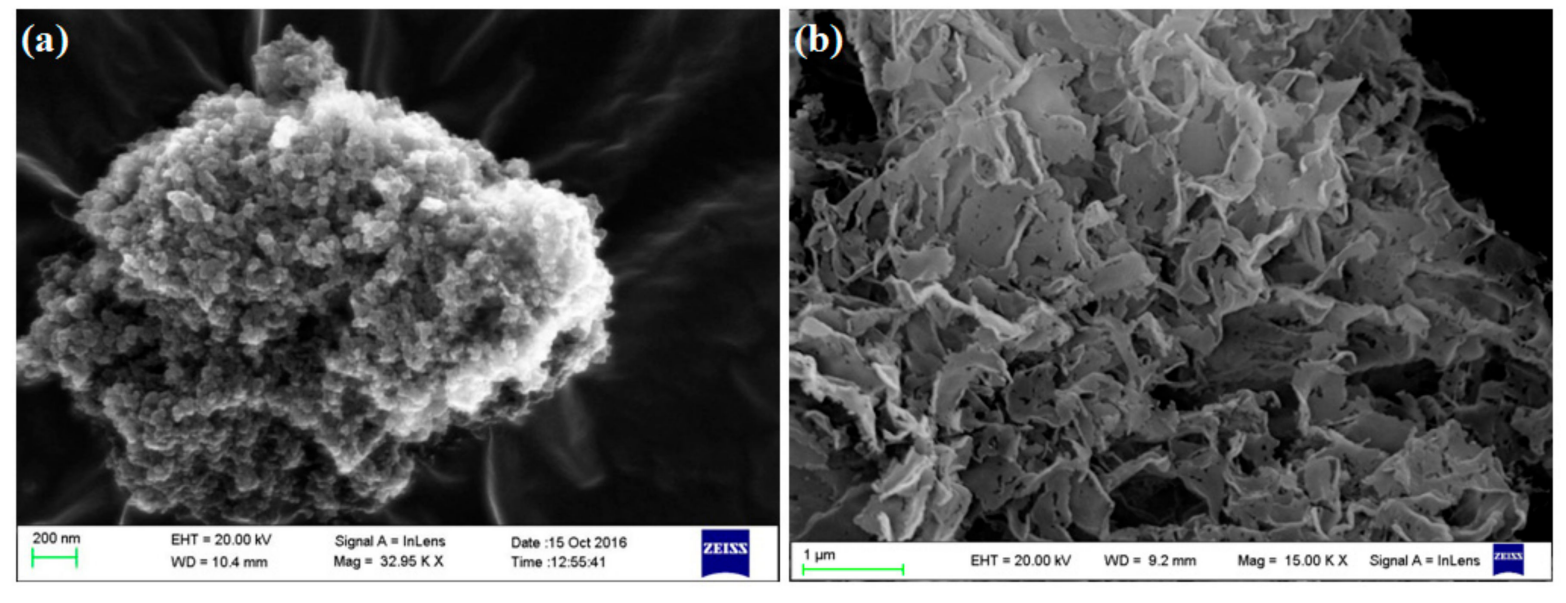

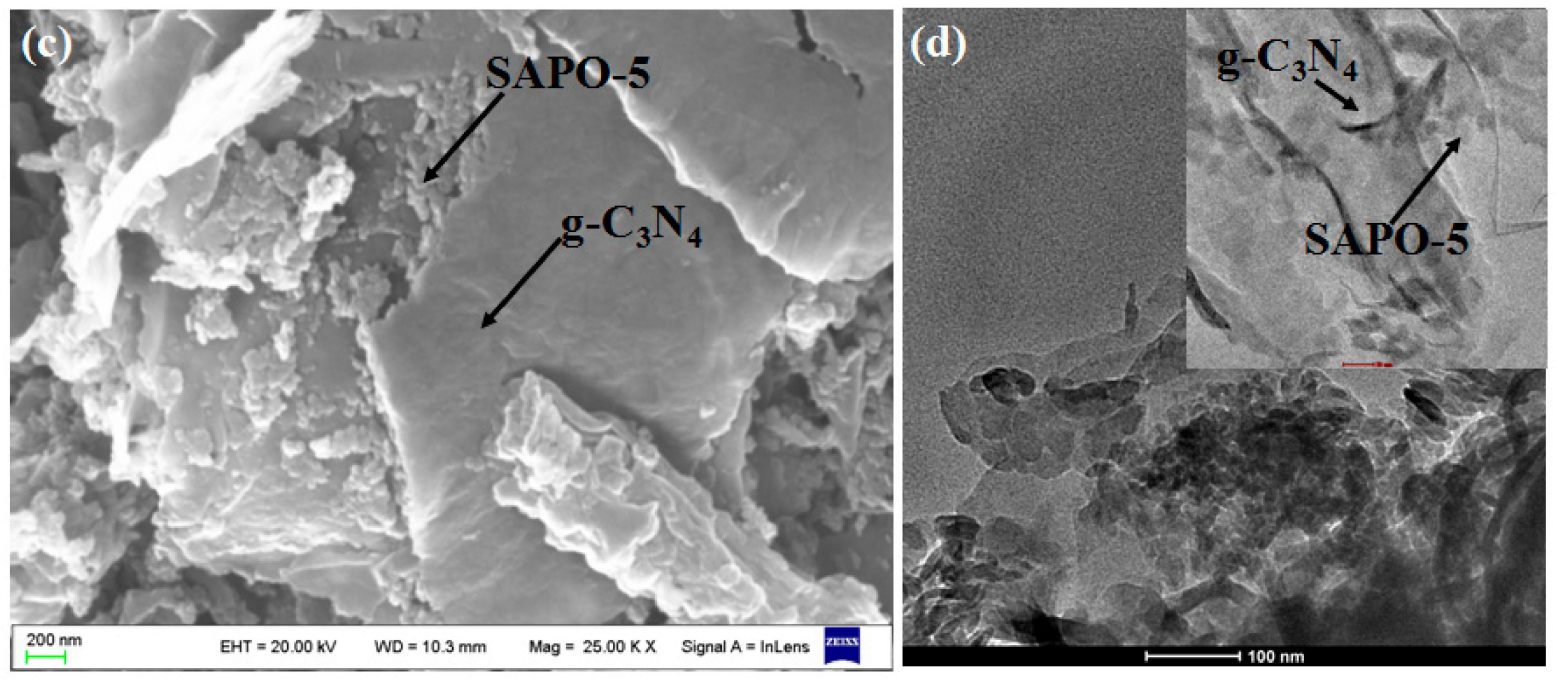
SEM images of pure SAPO-5 (**a**), pure g-C_3_N_4_ (**b**), and SAPO-5/g-C_3_N_4_-4 composite (**c**); and the TEM image of SAPO-5/g-C_3_N_4_-4 (**d**) (the inset in (**d**) is the TEM image under higher resolution).

Figure 3 shows SEM images of pure SAPO-5 and pure g-C_3_N_4_ (Figure 3a,b), and SEM image and TEM images of SAPO-5/g-C_3_N_4_ (Figure 3c,d), respectively. Obviously, SAPO-5 nanoparticles, with a particle size of 30–60 nm spread all over the g-C_3_N_4_nanosheets, can effectively retard the aggregation of g-C_3_N_4_. The TEM image displays the intimate contact between nanometer-sized SAPO-5 particles and ultrathin transparent g-C_3_N_4_, which indicates the formation of a hybrid structure facilitating and improving the photocatalytic activity of g-C_3_N_4_.

**Figure 4:**
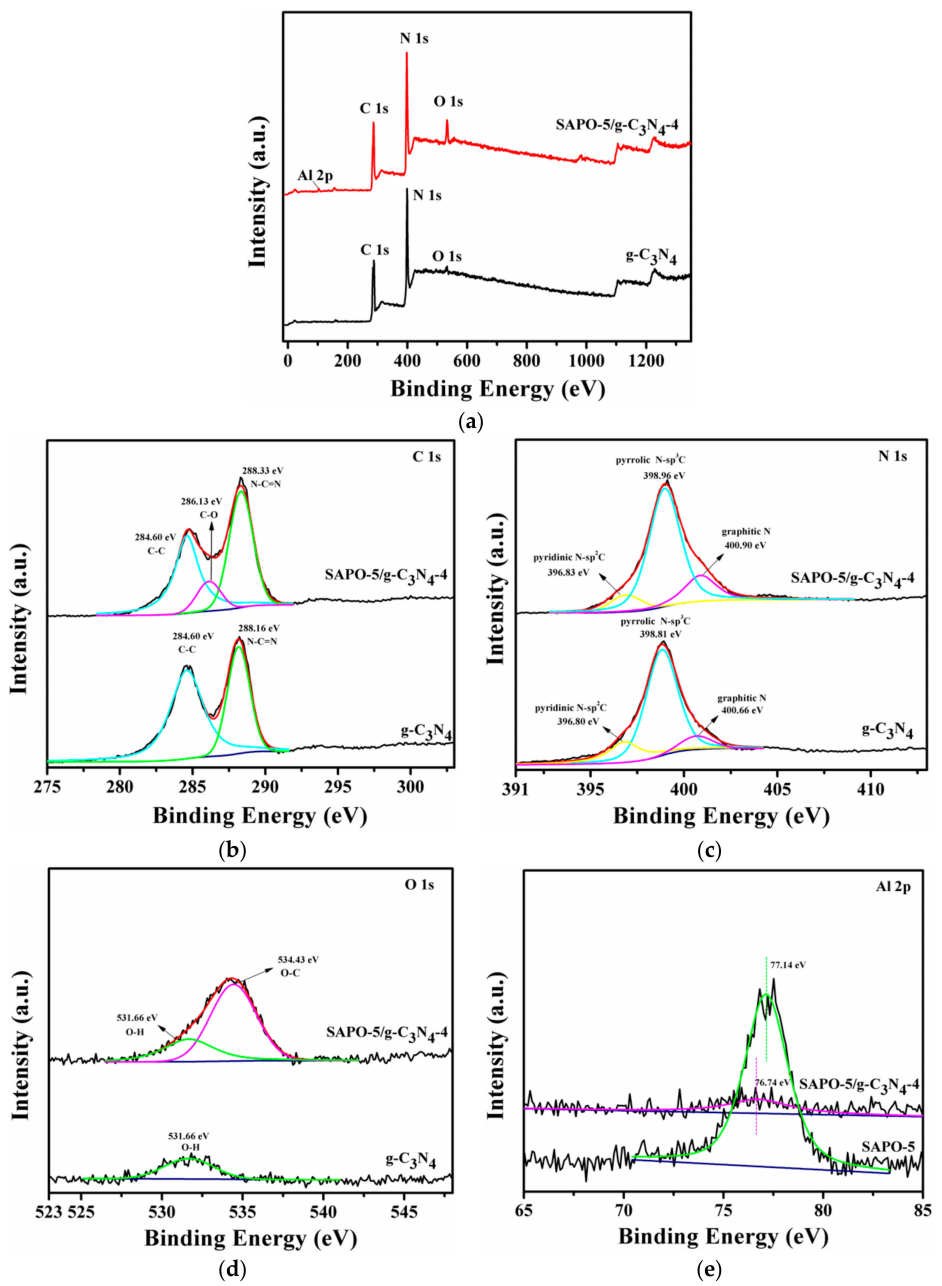
X-ray photoelectron spectroscopy (XPS) spectra of the survey (**a**), C 1s (**b**), N 1s (**c**), and O 1s (**d**) of pure g-C_3_N_4_ and SAPO-5/g-C_3_N_4_-4, and XPS spectra of Al 2p (**e**), pure SAPO-5, and SAPO-5/g-C_3_N_4_-4.

The chemical bonds in pure g-C_3_N_4_ and SAPO-5/g-C_3_N_4_-4 were measured by X-ray photoelectron spectroscopy (XPS), and the results were illustrated in Figure 4. The full spectrum (Figure 4a) indicated that C, N, O, and Al from SAPO-5 exist in the sample. Deeper analysis for heterojunction-like construction was carried out based on the spectra for C 1s, N 1s, O 1s, and Al 2p. All the peaks were calibrated by C 1s peak at 284.6 eV. For C 1s spectra (Figure 4b), compared with pure g-C_3_N_4_, the formation of C–O located at 286.1 eV of SAPO-5/g-C_3_N_4_-4 indicated the chemical contact between g-C_3_N_4_ and SAPO-5. Moreover, from the spectra of C 1s, N 1s, and O 1s it was obvious that C–C, N–C=N, pyridine N, pyrrole N, graphitic N, and O-H bonds existed in pure g-C_3_N_4_ and SAPO-5/g-C_3_N_4_-4 [24]. Furthermore, the binding energies of these bonds were all higher in SAPO-5/g-C_3_N_4_-4, which showed that the electron cloud density was lower in g-C_3_N_4_ and in SAPO-5/g-C_3_N_4_-4 compared with pure g-C_3_N_4_. In addition, the Al 2p binding energy in SAPO-5/g-C_3_N_4_-4 was lower than that in pure SAPO-5, which indicated that the electron cloud density in SAPO-5/g-C_3_N_4_-4 was higher than that in pure SAPO-5. Based on the above analysis, we can estimate that the photo-induced electrons move from g-C_3_N_4_ to the Al sites in SAPO-5 for SAPO-5/g-C_3_N_4_-4, which should facilitate the separation of photo-induced electron-hole pairs in g-C_3_N_4_.

**Figure 5:**
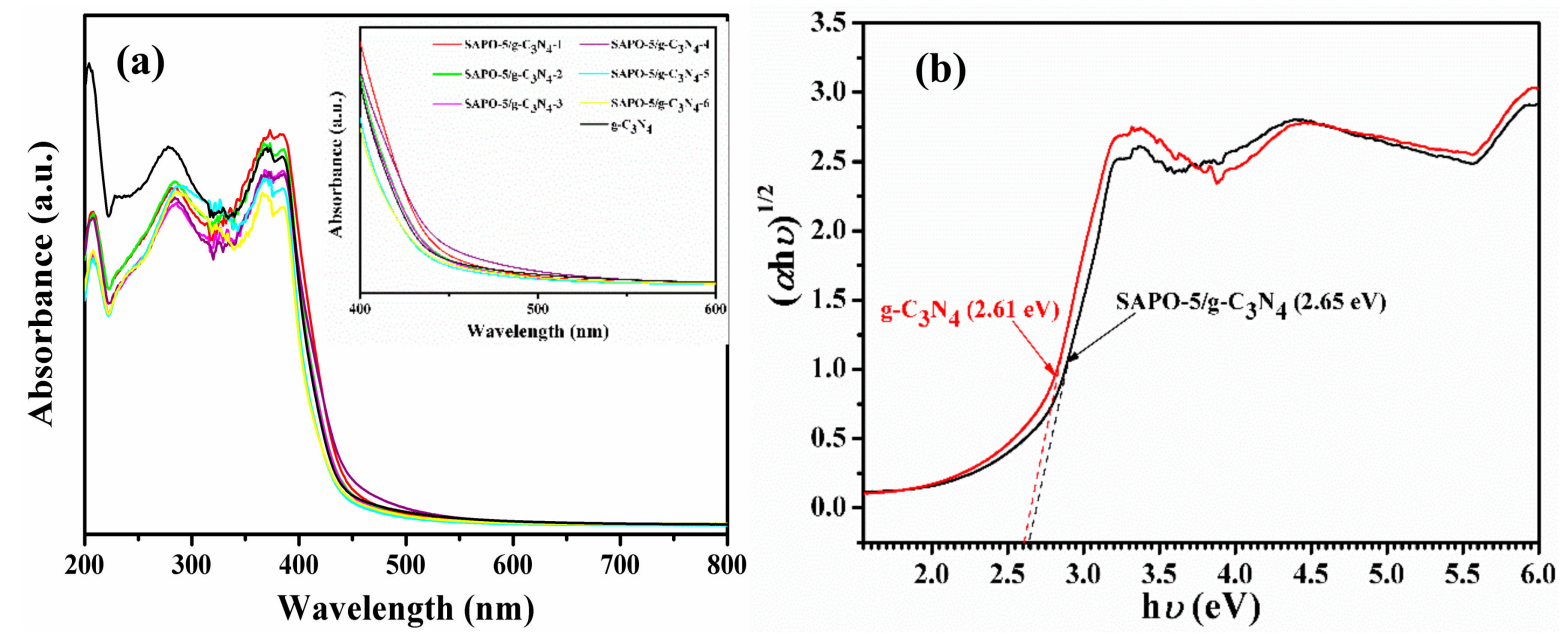
UV-vis diffuse reflection spectra (DRS) of pure g-C_3_N_4_ and SAPO-5/g-C_3_N_4_ composites with different SAPO-5 incorporation proportions (**a**), and plots of (αh*ν*)^1/2^ versus h*ν* for the band gap energy of pureg-C_3_N_4_ and SAPO-5/g-C_3_N_4_-4 (**b**).

The diffuse reflectance spectra (DRS) within a wavelength range of 200 nm to 800 nm for the synthesized composites and pure g-C_3_N_4_ are shown in Figure 5a. Obviously, the adsorption edges of the samples were similar, but when incorporating a small amount of SAPO-5 particles, the composites showed higher absorbance in the visible light range (inset in Figure 5a). When the amount of SAPO-5 was higher than 0.5 g, the absorbance decreased compared with pure g-C_3_N_4_. Obviously, a certain amount of SAPO-5 particles inhibited the aggregation of g-C_3_N_4_ effectively, which improved the absorption ability of visible light in g-C_3_N_4_. As the amount increased, the insulation property of SAPO-5 limited the absorption ability of visible light in g-C_3_N_4_ undesirably. In addition, band gaps of the samples were calculated according to the Tauc plot (where α, h, and *ν* were the linear absorption coefficient, Planck constant, and light frequency, respectively) [25]. The constant of 1/2 was adopted for the samples. Figure 5b shows a band gap comparison between pure g-C_3_N_4_ (2.61 eV) and SAPO-5/g-C_3_N_4_-4 (2.65 eV). Due to the dispersion of SAPO-5, the band gap of synthesized composites became larger, which should be attributed to the large band gap for most zeolites. Nevertheless, it demonstrated that the hybrid structures of SAPO-5/g-C_3_N_4_ have been formed successfully.

**Figure 6:**
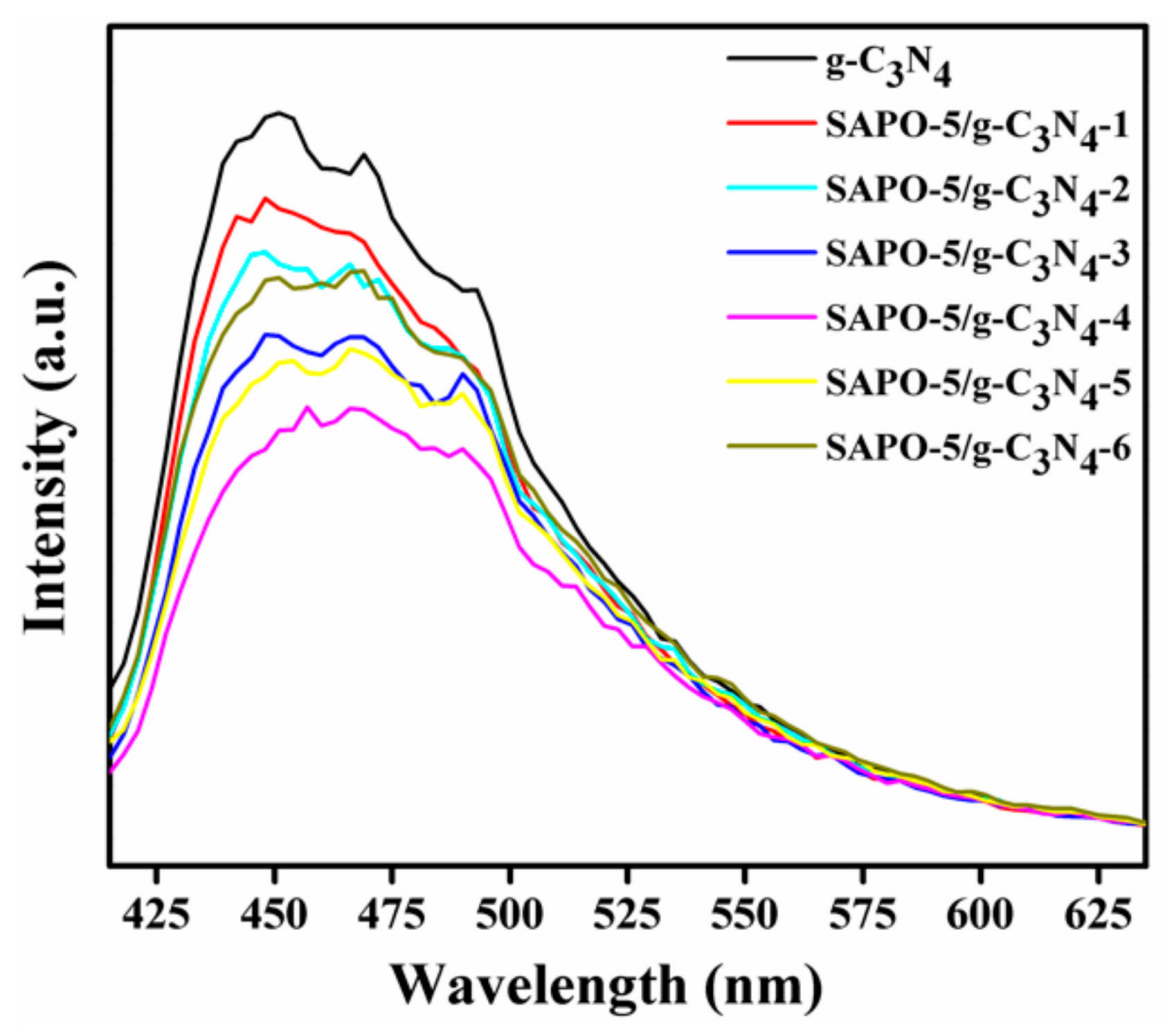
The photoluminescence (PL) spectra of pure g-C_3_N_4_ and SAPO-5/g-C_3_N_4_ composites with different SAPO-5 incorporation proportions.

Photoluminescence (PL) at room temperature is always applied when evaluating the charge recombination rate of photocatalysts. Higher intensity means a faster charge recombination rate, in theory [18]. Figure 6 indicates that after adding SAPO-5, the photo-induced electron-hole pairs of g-C_3_N_4_ can be separated much more effectively. As expected, the separation efficiency was affected by the incorporation proportion of SAPO-5. When the incorporation proportion was 0.5 g, g-C_3_N_4_/SAPO-5 showed the most preferable charge transferability, which further verified the formation of a hybrid structure of SAPO-5/g-C_3_N_4_.

**Figure 7:**
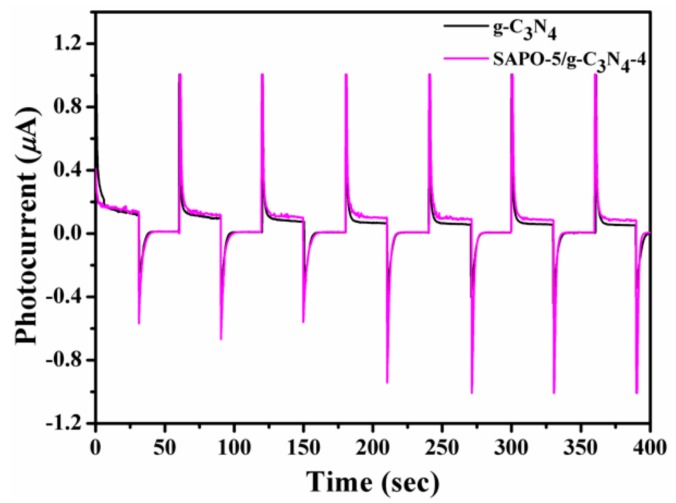
Thephotocurrent patterns of pure g-C_3_N_4_ and SAPO-5/g-C_3_N_4_-4.

It is meaningful to use photocurrent to evaluate the photo-induced electron density of activated photocatalysts. As shown in Figure 7, compared to pure g-C_3_N_4_, it was obvious that SAPO-5/g-C_3_N_4_ had a stronger electric current when the light was turned on. Naturally, the electric current decreased sharply and disappeared finally when the light was turned off.

**Figure 8:**
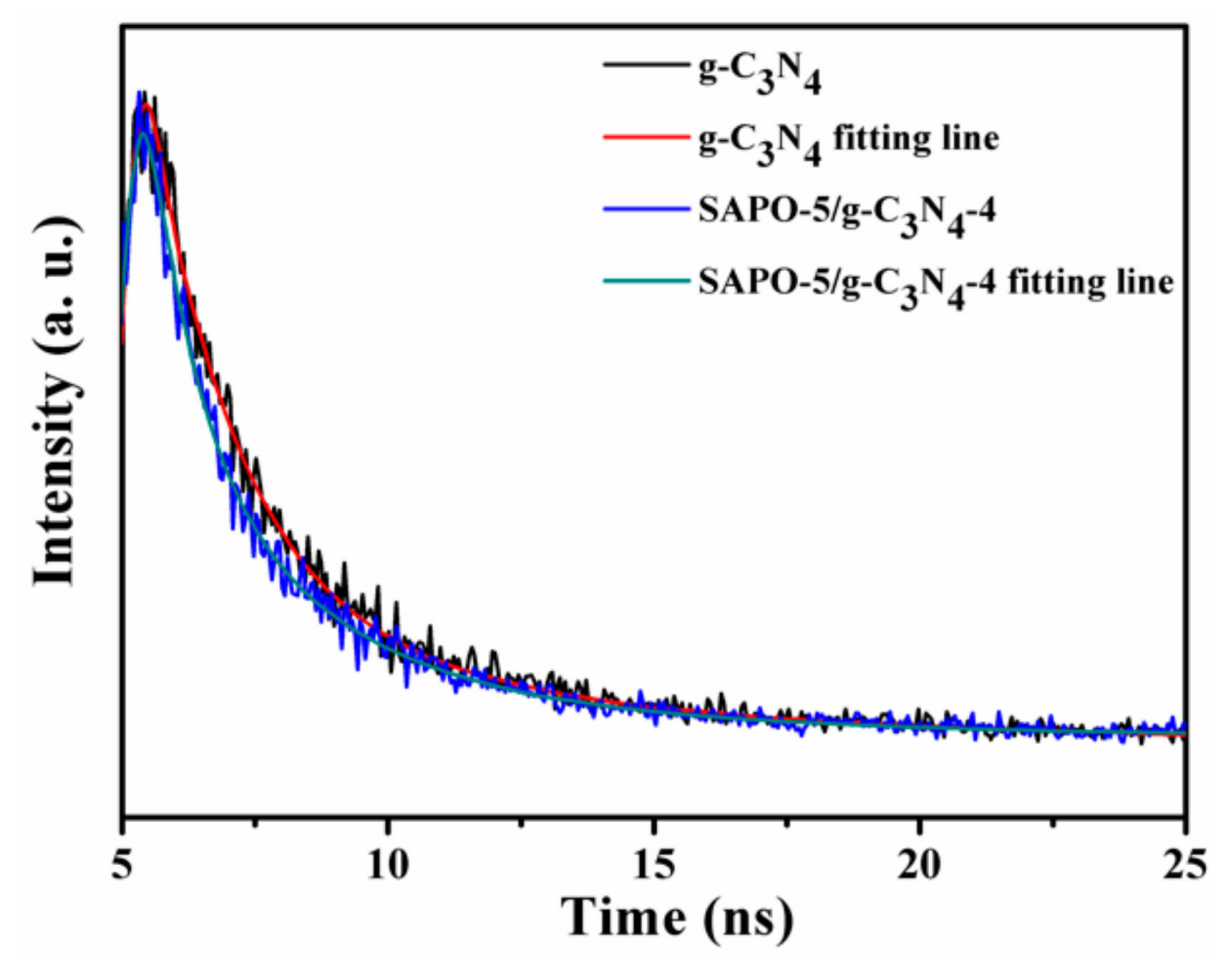
The time-resolved fluorescence decay spectra of pure g-C_3_N_4_ and SAPO-5/g-C_3_N_4_-4.

The separation efficiency of photo-induced carriers is one of the most critical issues during the investigation of photocatalysts. Time-resolved fluorescence was applied when recording the photo-induced carrier separation processes of pure g-C_3_N_4_ and SAPO-5/g-C_3_N_4_-4 (Figure 8). The fluorescence times of pure g-C_3_N_4_ and SAPO-5/g-C_3_N_4_ were 3.0 ns and 4.4 ns, respectively. As discussed in our previous publication, SAPO-5 can hardly be activated under 380 nm [12], thus, we can suppose that SAPO-5/g-C_3_N_4_-4 showed a much higher photo-induced carrier separation rate than pure g-C_3_N_4_. This should have made many contributions to the photocatalytic performance of the samples.

### 3.2. Visible-Light Photocatalytic Performance

The photocatalytic activity of catalysts is affected by many factors. Herein, we first discussed the role of dye concentration in the photocatalytic process of the samples to explore an appropriate environment reflecting the higher photocatalytic potential of photocatalysts.

#### 3.2.1. Effect of Initial RhB Concentration

**Figure 9:**
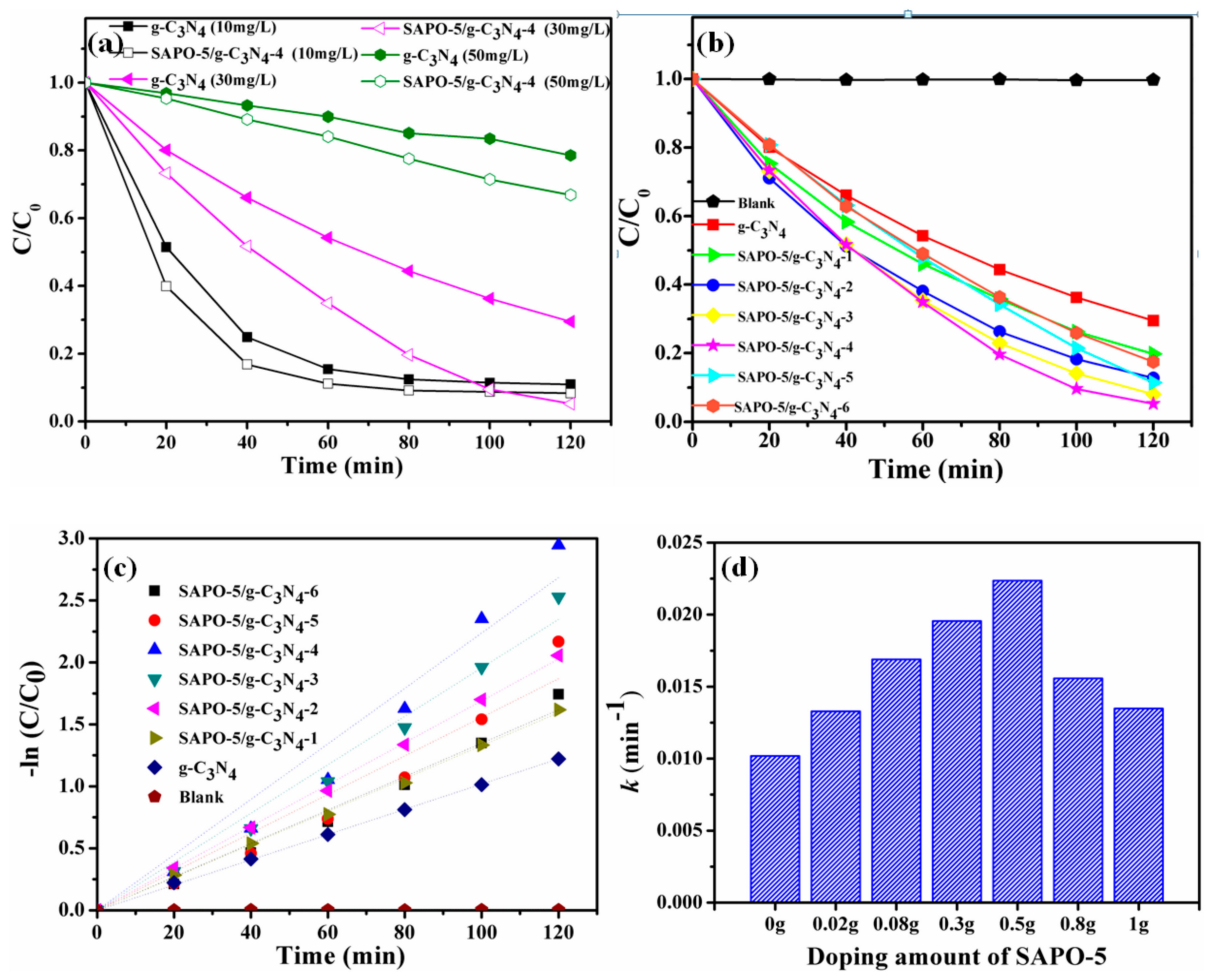
The photocatalytic performances for RhB degradation in the presence of g-C_3_N_4_ and SAPO-5/g-C_3_N_4_-4 under different RhB concentrations (**a**) ([RhB]_initial_: 10–50 mg/L, catalyst dose: 50 mg, volume: 50 mL, reaction time: 120 min, light source: Xenon lamp, 500 W, λ > 420 nm), photocatalytic performances (**b**), pseudo-first-order kinetic plots (**c**), and reaction rate constant (**d**) for RhB degradation in the presence of g-C_3_N_4_ and SAPO-5/g-C_3_N_4_ composites with different SAPO-5 incorporation proportions ([RhB]_initial_: 30 mg/L, catalyst dose: 50 mg, volume: 50 mL, reaction time: 120 min, light source: Xenon lamp, 500 W, λ > 420 nm).

The initial RhB concentrations were fixed on 10 mg/L, 30 mg/L, and 50 mg/L, respectively. As shown in Figure 9a, the photocatalytic activities of pure g-C_3_N_4_ and composite SAPO-5/g-C_3_N_4_-4 were affected significantly as the concentration increased, especially when RhB concentration was 50 mg/L. For pure g-C_3_N_4_, the degradation efficiency decreased from 89.05% to 21.45% sharply. However, for SAPO-5/g-C_3_N_4_-4, the degradation efficiencies were similar under the RhB concentrations of 10 mg/L (91.63%) and 30 mg/L (94.74%), while it decreased to 33.12% when the RhB concentration increased to 50 mg/L. In general, photocatalysts with the same amount of active sites can’t decompose too many extra organic composites in higher RhB concentration, which was in accordance with the behavior of pure g-C_3_N_4_. In comparison, composite SAPO-5/g-C_3_N_4_-4 still behaved well in RhB with a higher concentration of 30 mg/L, which was likely due to the sufficient amount of active sites which were superfluous in the RhB solution with a small concentration of 10 mg/L. Thus, 30 mg/L was an optimum concentration to exhibit the superior photocatalytic activity of SAPO-5/g-C_3_N_4_-4 compared to pure g-C_3_N_4_. The corresponding plots obtained from the UV-vis spectrophotometer can be seen in Figure S1.

#### 3.2.2. Effect of the SAPO-5 Incorporation Proportion

After optimizing the proper RhB concentration, the best SAPO-5 incorporation proportion was screened for SAPO-5/g-C_3_N_4_ to exhibit higher RhB degradation performance. As shown in Figure 9b, the RhB degradation efficiencies increased remarkably after they were incorporated with SAPO-5. When the SAPO-5 incorporation proportion is 0.5 g, the degradation efficiency increased from 70.52% to 94.74%, which is 34.34% higher than pure g-C_3_N_4_. To confirm that RhB didn’t degrade itself, a blank experiment was carried out and the degradation efficiency was only 0.27%. In addition, Figure 9c,d indicated that the degradation rate constant increased from 0.013 min^−1^ to 0.022 min^−1^ compared with pureg-C_3_N_4_ (Table S2). The corresponding plots obtained from the UV-vis spectrophotometer can also be seen in Figure S2.

To further evaluate the photocatalytic activity quantitatively, it is necessary to analyze the synergistic factor (SF) in the composites, which is based on the following equation [26]:

(1) $$\mathrm{SF}=\frac{k_{g-C3N4/\mathrm{SAPO}-5\left(X:Y\right)}}{k_{\mathrm{SAPO}-5}\times \left(1-X\right)+k_{g-C3N4}\times X}$$

where *k*_g-C3N4/SAPO-5_, *k*_SAPO-5_, *k*_g-C3N4_ are the photodegradation rates of SAPO-5/g-C_3_N_4_, SAPO-5, and pure g-C_3_N_4_ for RhB degradation (30 mg/L) under visible illumination (Figure 9d). X and Y are the contents of SAPO-5 and pure g-C_3_N_4_ in the SAPO-5/g-C_3_N_4_ composites (Table S1). Obviously, *k*_SAPO-5_ is regarded as zero because SAPO-5 can hardly have photocatalytic activity (Table S2). All the composites show a synergistic effect because the synergistic factors are all larger than 1.00. Moreover when SAPO-5 content is 30.25%, SAPO-5/g-C_3_N_4_ shows the greatest synergistic effect leading to the highest RhB degradation in this work.

### 3.3. Reusability

**Figure 10:**
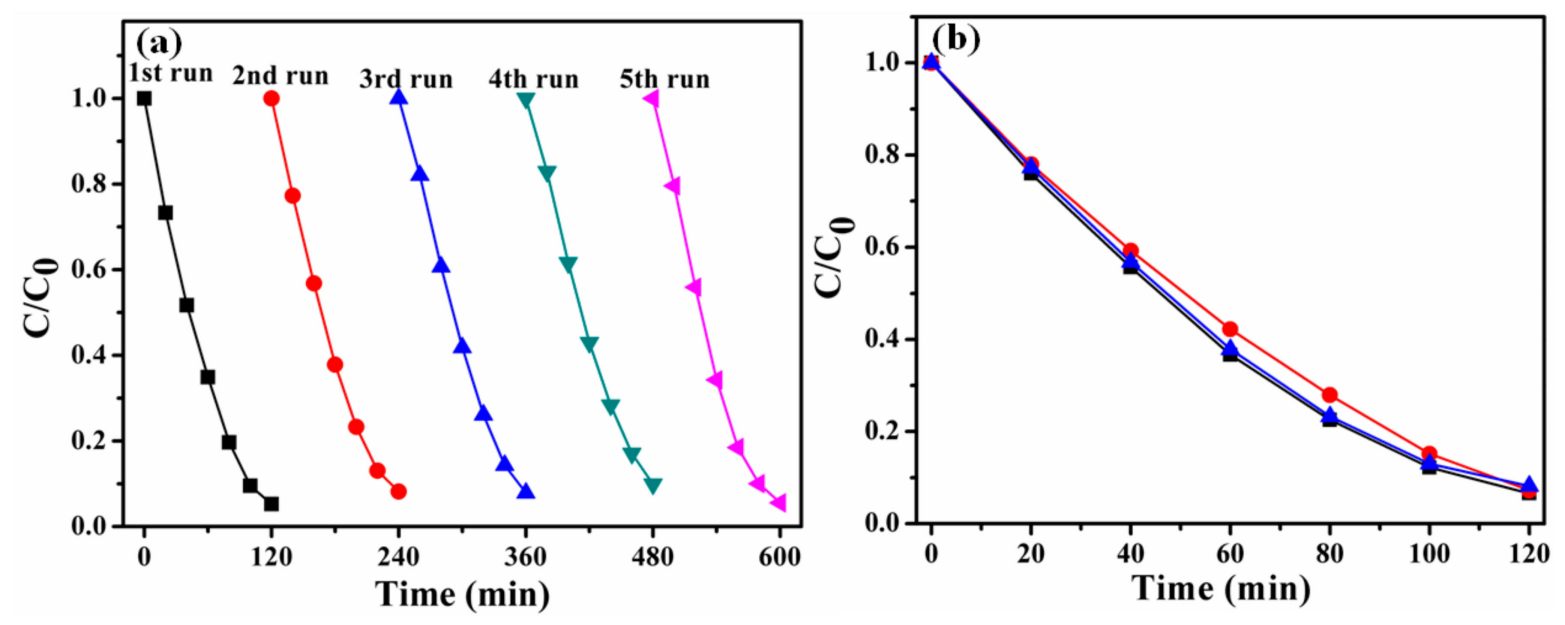
The recycling results of the photocatalytic performance of SAPO-5/g-C_3_N_4_-4 for RhB degradation (**a**) and the stability of preparing SAPO-5/g-C_3_N_4_-4 evaluated by RhB degradation (**b**) ([RhB]_initial_: 30 mg/L, catalyst dose: 50 mg, volume: 50 mL, reaction time: 120 min, light source: Xenon lamp, 500 W, λ > 420 nm).

Five-time cyclic experiments have been carried out to confirm the stability of SAPO-5/g-C_3_N_4_-4. As shown by Figure 10, it turned out that SAPO-5/g-C_3_N_4_-4 showed good stability during RhB degradation under visible light illumination. To further demonstrate that conclusion, SAPO-5/g-C_3_N_4_-4, after the 5th recycling, was characterized by XRD and SEM (Figures S3 and S4). As expected, both the crystal phase and microstructure remained unchanged during the whole experiment.

Moreover, synthesis reproducibility of photocatalysts is critical for practical application. SAPO-5/g-C_3_N_4_-4 have been synthesized several times and applied to the photodegradation for RhB. As Figure 10b showed, this sample can easily be synthesized and shows similar photocatalytic activity, which indicates the ideal synthesis reproducibility of SAPO-5/g-C_3_N_4_-4.

### 3.4. Synergistic Photocatalytic Degradation Mechanism

**Figure 11:**
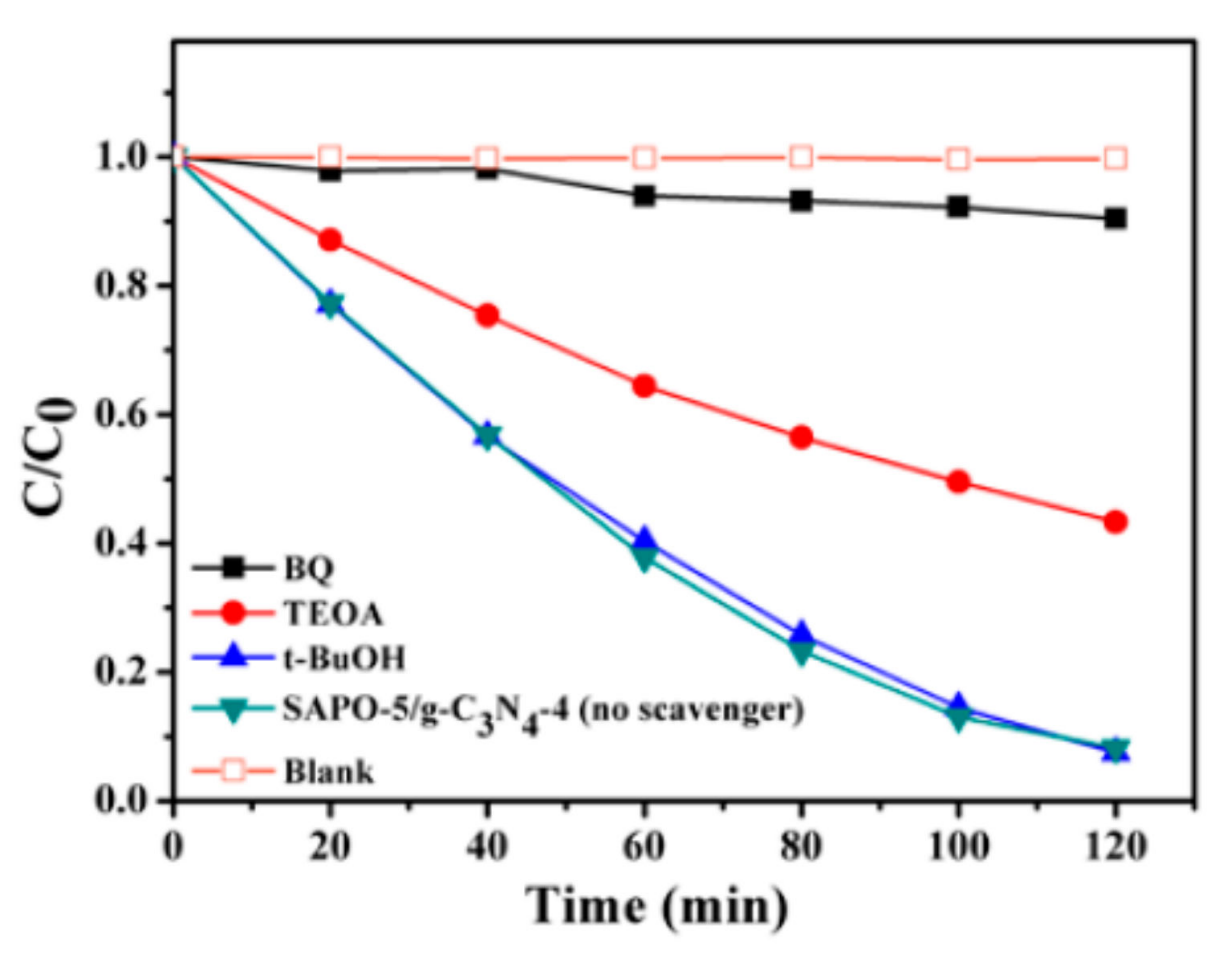
The comparison of the photocatalytic activities of SAPO-5/g-C_3_N_4_-4 for the degradation of RhB with the addition of BQ, TEOA, t-BuOH, or without scavengers.

Investigation on the crucial active species during the photocatalytic process using as-synthesized SAPO-5/g-C_3_N_4_-4 is very important for proposing the photocatalytic degradation mechanism. Trapping agents—1.4-benzoquinone (BQ), triethanolamine (TEOA), and tertiary butanol (t-BuOH)—were used for demonstrating the significances during the photocatalytic reaction of the active species—superoxide radical (•O_2_^−^), hole (h^+^), and hydroxide radical (•OH)—respectively. As shown in Figure 11, it is obvious that •OH doesn’t make any contribution to the photocatalytic performance. Compared with the result without any trapping agents, •O_2_^−^ has a more significant role than h^+^.

SAPO-5/g-C_3_N_4_-4 + h*ν*→SAPO-5/g-C_3_N_4_-4* (2)

O_2_ + e^−^ (Al^2+^)→•O_2_^−^ (3)

•O_2_^−^ + RhB→Colourless Products (4)

RhB + h^+^→Colourless Products (5)

**Scheme 1:**
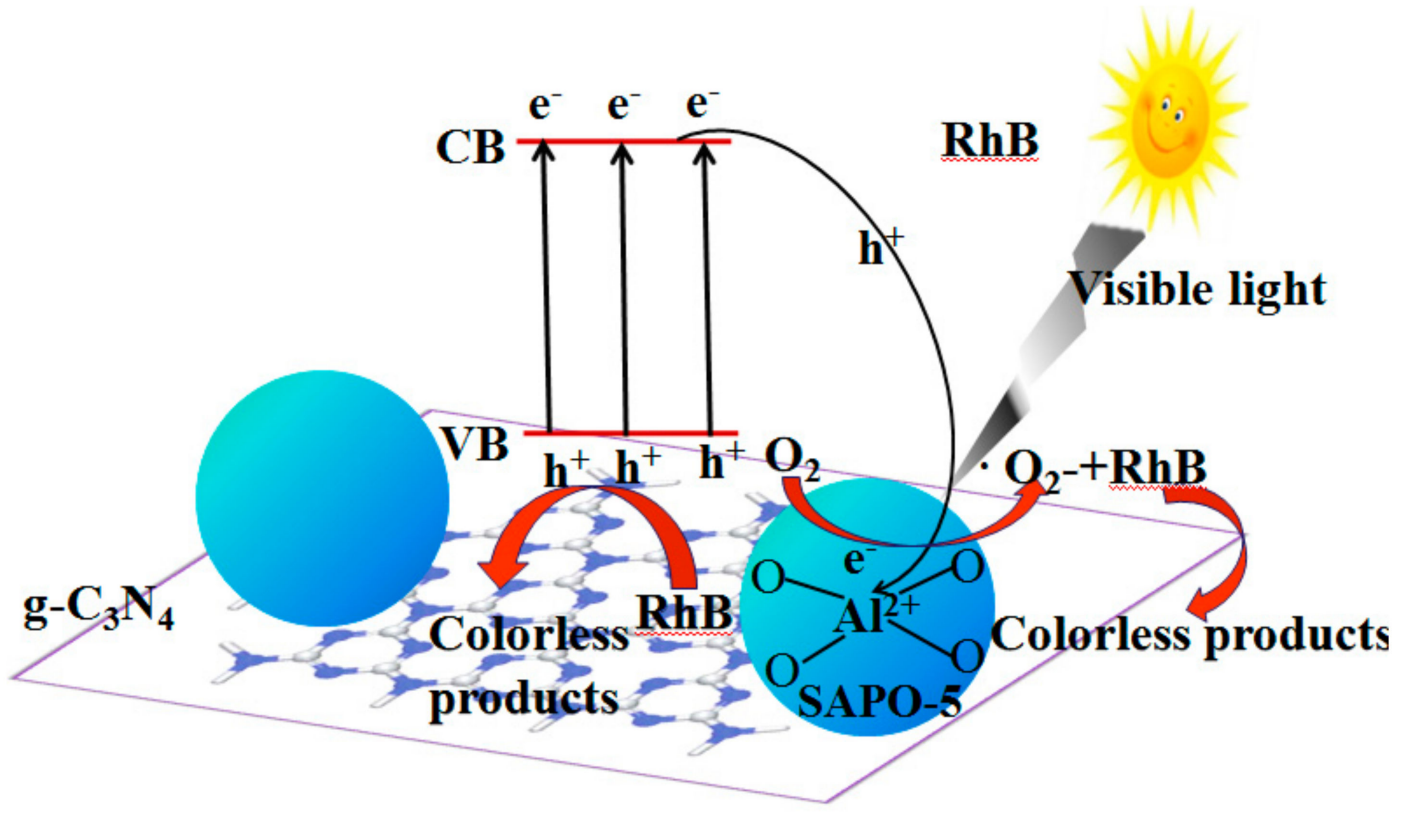
The proposed scheme for the separation and transfer of photogenerated charges in SAPO-5/g-C_3_N_4_-4.

The photocatalytic mechanism can be described by Equations (2)–(5), which will be illustrated in detail, as follows. For g-C_3_N_4_, the electron transmission from the valence band (VB) to the conduct band (CB) will easily happen under visible-light illumination. After SAPO-5/g-C_3_N_4_-4 composite forms, along with the band gap restructuring, SAPO-5 can also be activated and [Al^2+^–O^−^]* appeared. As described in our previous research, SAPO-5 becomes both of an electron acceptor and an electron donor [12]. The photo-induced electron from the conduction band g-C_3_N_4_ will migrate to the activated Al sites of SAPO-5, which facilitates the photo-induced electron-hole separation of g-C_3_N_4_. That is in accordance with the mechanism of the zeolite Y based TiO_2_photocatalyst [27]. Thus, the photocatalytic reaction proceeded at the valence band (VB) of g-C_3_N_4_ and Al sites in the SAPO-5 framework (Scheme 1). Based on the capture experiments, we can estimate that the RhB molecules were oxidized by the h^+^ of g-C_3_N_4_ and reduced by the •O_2_^−^ formed by capturing O_2_ at Al sites in SAPO-5, respectively.

### 3.5. Comparison

To further evaluate the significance of the SAPO-5/g-C_3_N_4_ designed in this work, the photocatalytic performance of SAPO-5/g-C_3_N_4_ was compared with other g-C_3_N_4_-based composites applied for organic pollutants (especially for RhB) degradation (Table S3). Considering both the degradation efficiency and apparent rate, SAPO-5/g-C_3_N_4_ showed promising photocatalytic activity. It provides proof for a bright future of constructing zeolitic material-modified g-C_3_N_4_ composites in environmental purification.

## 4. Conclusions

In summary, we have successfully prepared SAPO-5/g-C_3_N_4_ heterojunction-like composites. The effects of the SAPO-5 incorporation proportion in SAPO-5/g-C_3_N_4_ and pristine RhB concentration on the photocatalytic performance of SAPO-5/g-C_3_N_4_ has been discussed in detail. As a result, the RhB (30 mg/L) degradation efficiency under the visible light illumination of optimized SAPO-5/g-C_3_N_4_ is improved by 40.6% compared with that of pure g-C_3_N_4_. The photocatalytic mechanism of SAPO-5/g-C_3_N_4_ heterojunction has been proposed. This significant enhancement should be attributed to the heterojunction-like contact between SAPO-5 and g-C_3_N_4_, which promotes the separation of photo-induced electron-hole pairs in g-C_3_N_4_. This work opens up new prospects for exploring novel g-C_3_N_4_/SAPOs heterojunction-like composites with a much higher visible-light photocatalytic performance.

## Supplementary Materials

The following are available online at https://www.mdpi.com/1996-1944/12/23/3948/s1, Figure S1: UV-vis of RhB degradation by g-C_3_N_4_ and SAPO-5/g-C_3_N_4_-4 ([RhB]_initial_: 10–50 mg/L, catalyst dose: 50 mg, volume: 50 mL, reaction time: 120 min, light source: Xenon lamp, 500 W, λ > 420 nm), Figure S2: UV-vis of RhB degradation by g-C_3_N_4_ and SAPO-5/g-C_3_N_4_ composites with different SAPO-5 doping amount ([RhB]_initial_: 30 mg/L, catalyst dose: 50 mg, volume: 50 mL, reaction time: 120 min, light source: Xenon lamp, 500 W, λ > 420 nm), Figure S3: XRD patterns for fresh SAPO-5/g-C_3_N_4_-4 and SAPO-5/g-C_3_N_4_-4 after 5th recycling, Figure S4: SEM image of SAPO-5/g-C_3_N_4_-4 after 5th recycling. Table S1: g-C_3_N_4_ and SAPO-5 contents in precursors and SAPO-5/g-C_3_N_4_ composites, Table S2: Degradation rate constant and cofactor of SAPO-5/g-C_3_N_4_ samples compared with pure g-C_3_N_4_, Table S3: Comparison of the photocatalytic dye degradation performance for g-C_3_N_4_-based binary photocatalysts under visible illumination.
