# Supplementary material for: Enhanced Visible-Light Photocatalytic Performance of SAPO-5-Based g-C3N4 Composite for Rhodamine B (RhB) Degradation
Source: Materials (Basel). 2019 Nov 28;12(23):3948. doi: 10.3390/ma12233948 (PMC6926513; doi:10.3390/ma12233948)
Supplement: Supplementary file 1 [file materials-12-03948-s001.pdf]

materials

Supporting Information: Enhanced Visible-light Photocatalytic Performance of SAPO-5-based g-C_3N_4 Composite for Rhodamine (RhB) Degradation

Lingfang Qiu [1], Zhiwei Zhou [1], Mengfan Ma [1], Ping Li [1], Jinyong Lu [1],Yingying Hou [1], Xiangshu Chen [2] and Shuwang Duo [1,*]

[1] Jiangxi Key Laboratory of Surface Engineering, Jiangxi Science and Technology Normal University, Nanchang 330013, China
[2] Institute of Advanced Materials (IAM), State-Province Joint Engineering Laboratory of Zeolite Membrane Materials, College of Chemistry and Chemical Engineering, Jiangxi Normal University, Nanchang, 330022, China
* Correspondence: swduo@imr.ac.cn; Tel.: +86 79183831266; Fax: +86 79183831266.

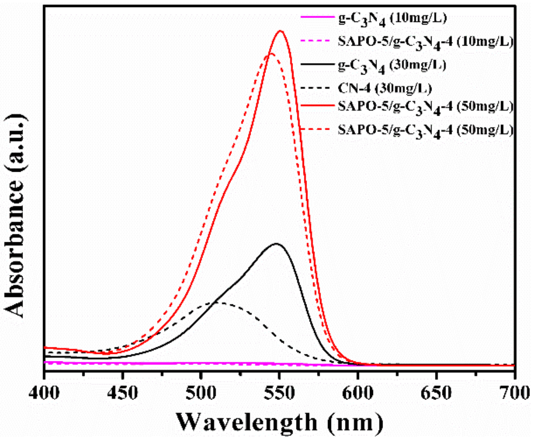

Figure S1. UV-vis of RhB degradation by g-C_3N_4 and SAPO-5/g-C_3N_4-4 ($[RhB]_{initial}$: 10–50 mg/L, catalyst dose: 50 mg, volume: 50 mL, reaction time: 120 min, light source: Xenon lamp, 500 W, $\lambda > 420$ nm).

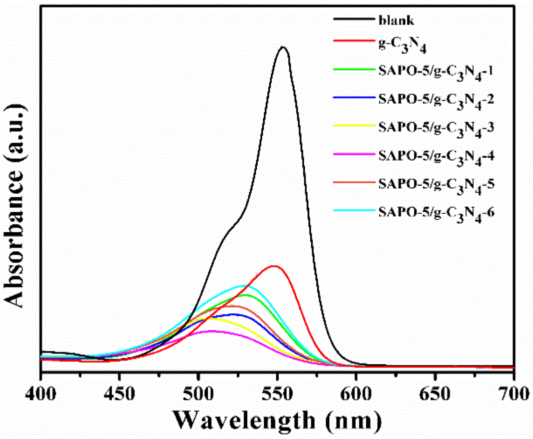

Figure S2. UV-vis of RhB degradation by g-C_3N_4 and SAPO-5/g-C_3N_4 composites with different SAPO-5 doping amount ($[RhB]_{initial}$: 30 mg/L, catalyst dose: 50 mg, volume: 50 mL, reaction time: 120 min, light source: Xenon lamp, 500 W, $\lambda > 420$ nm).

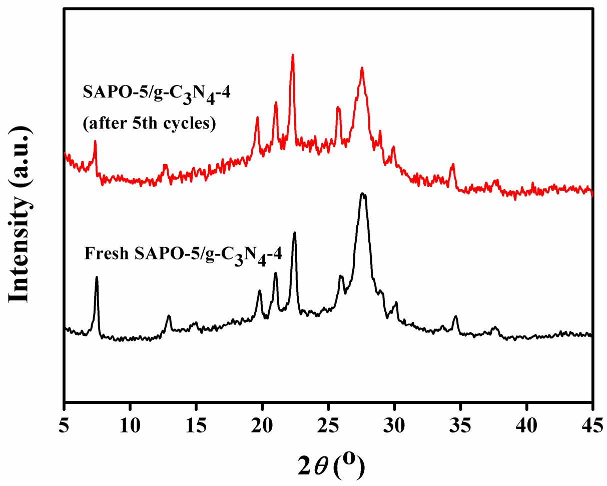

Figure S3. XRD patterns for fresh SAPO-5/g-C_3N_4-4 and SAPO-5/g-C_3N_4-4 after 5th recycling.

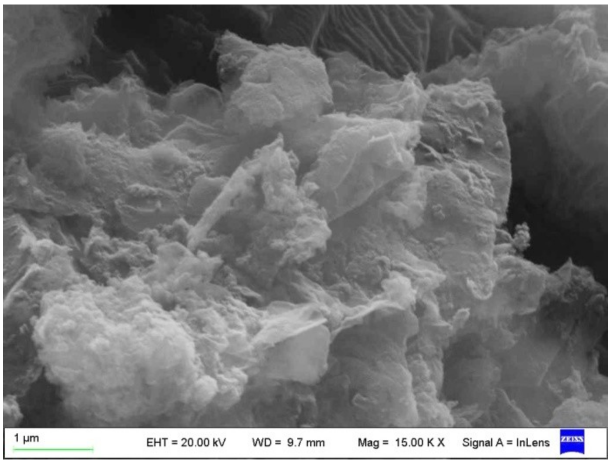

Figure S4. SEM image of SAPO-5/g-C_3N_4-4 after 5th recycling.

Table S1. g-C_3N_4 and SAPO-5 contents in precursors and SAPO-5/g-C_3N_4 composites.

Sample Name	SAPO-5 (g)	W_{SAPO-5} (wt.%) [a]	SAPO-5:g-C_3N_4 [b]	Y_{SAPO-5} (wt.%) [c]	X_{g-C3N4} (wt.%) [d]
g-C_3N_4	0	0	0	0	100
SAPO-5/g-C_3N_4-1	0.02	0.11	0.03	1.70	98.30
SAPO-5/g-C_3N_4-2	0.08	0.44	0.07	6.48	93.52
SAPO-5/g-C_3N_4-3	0.3	1.64	0.26	20.64	79.36
SAPO-5/g-C_3N_4-4	0.5	2.70	0.43	30.25	69.75
SAPO-5/g-C_3N_4-5	0.8	4.26	0.69	40.96	59.04
SAPO-5/g-C_3N_4-6	1.0	5.26	0.87	46.45	53.55

Note: [a] mass ratio of SAPO-5 in whole precursor; [b] mass ratio of SAPO-5 to g-C_3N_4; [c] mass ratio of SAPO-5 in product; [d] mass ratio of g-C_3N_4 in product.

Table S2. Degradation rate constant and cofactor of SAPO-5/g-C_3N_4 samples compared with pure g-C_3N_4.

Sample name	Degradation rate k (min^{-1}) [a]	Synergistic Factor (SF)
g-C_3N_4	0.007	-
SAPO-5/g-C_3N_4-1	0.013	2.07
SAPO-5/g-C_3N_4-2	0.017	2.77
SAPO-5/g-C_3N_4-3	0.020	3.77
SAPO-5/g-C_3N_4-4	0.022	4.91
SAPO-5/g-C_3N_4-5	0.016	4.04
SAPO-5/g-C_3N_4-6	0.013	3.85

Note: [a] Apparent rate for RhB with a concentration of 30 mg/L.

Table S3. Comparison of the photocatalytic dye degradation performance for g-C_3N_4-based binary photocatalysts under visible illumination.

Photocatalyst	Dosage (mg)	Dye Species	Dye Concentration (mg/L)	Volume of Dye Solution (mL)	Degradation Efficiency	Degradation Rate (min^{-1})	Reference
MnO_2/g-C_3N_4	50	RhB	10	50	91.30%	0.033	[1]
MoS_2/g-C_3N_4	30	RhB	20	50	98.20%	0.032	[2]
CNT/g-C_3N_4	50	MB	10	50	-	0.011	[3]
CQDs/g-C_3N_4	50	RhB	10	100	95.20%	0.014	[4]
ZnO/g-C_3N_4	50	MO	10	100	90.80%	-	[5]
BiOBr/g-C_3N_4	50	RhB	30	50	-	0.013	[6]
$LaFeO_3$/g-C_3N_4	20	RhB	15	100	-	0.013	[7]
UiO/g-C_3N_4	20	RhB	10	50	-	0.009	[8]
Ag_3VO_4/g-C_3N_4	50	Basic fuchsin	20	50	-	0.015	[9]
SnO_{2-x}/g-C_3N_4	100	RhB	10	100	-	0.088	[10]
SAPO-5/g-C_3N_4	50	RhB	30	50	94.74%	0.022	This work

References

1. Xia, P.; Zhu, B.; Bei, C.; Yu, J.; Xu, J., 2D/2D g-C_3N_4/MnO_2 nanocomposite as a direct Z-scheme photocatalyst for enhanced photocatalytic activity. *ACS Sustain. Chem. Eng.* **2018,** 6, (1), 965-973.
2. Wang, X.; Hong, M.; Zhang, F.; Zhuang, Z.; Yu, Y., Recyclable nanoscale zero valent iron doped g-C_3N_4/MoS_2 for efficient photocatalytic of RhB and Cr(VI) driven by visible light. *ACS Sustain. Chem. Eng.* **2016,** 4, (7), 4055-4063.
3. Yuanguo, X.; Hui, X.; Lei, W.; Jia, Y.; Huaming, L.; Yanhua, S.; Liying, H.; Guobin, C., The CNT modified white C_3N_4 composite photocatalyst with enhanced visible-light response photoactivity. *Dalton. T.* **2013,** 42, (21), 7604-7613.
4. Hong, Y.; Meng, Y.; Zhang, G.; Yin, B.; Yong, Z.; Shi, W.; Li, C., Facile fabrication of stable metal-free CQDs/g-C_3N_4 heterojunctions with efficiently enhanced visible-light photocatalytic activity. *Sep. Purif. Technol.* **2016**, *171*, 229–237.
5. Le, S.; Jiang, T.; Li, Y.; Qian, Z.; Li, Y.; Fang, W.; Ming, G., Highly efficient visible-light-driven mesoporous graphitic carbon nitride/ZnO nanocomposite photocatalysts. *Appl. Catal. B: Environ.* **2017**, *200*, 601–610.
6. Man, J.; Shi, Y.; Huang, J.; Lei, W.; Wang, Q., Synthesis of flower-like g-C_3N_4/BiOBr with enhanced visible light photocatalytic activity for degredation of dyes. *Eur. J. Inorg. Chem.* **2018,** 2018, (17), 1834-1841.
7. Qian, L.; Jie, J.; Liu, C.; Song, X.; Li, Z., Constructing a novel p-n heterojunction photocatalyst $LaFeO_3$/g-C_3N_4 with enhanced visible-light-driven photocatalytic activity. *J. Alloy. Compound.* **2017**, *709*, 542–548.
8. Zhang, X.; Yang, Y.; Huang, W.; Yang, Y.; Wang, Y.; He, C.; Liu, N.; Wu, M.; Tang, L., g-C_3N_4/UiO-66 nanohybrids with enhanced photocatalytic activities for the oxidation of dye under visible light irradiation. *Mater. Res. Bull.* **2018**, *99*, 349–358.
9. Wang, S.; Li, D.; Cheng, S.; Yang, S.; Yuan, G.; He, H., Synthesis and characterization of g-C_3N_4/Ag_3VO_4 composites with significantly enhanced visible-light photocatalytic activity for triphenylmethane dye degradation. *Appl. Catal. B: Environ.* **2014**, *144*, 885–892.
10. He, Y.; Zhang, L.; Fan, M.; Wang, X.; Walbridge, M. L.; Nong, Q.; Ying, W.; Zhao, L., Z-scheme SnO_2−x/g-C_3N_4 composite as an efficient photocatalyst for dye degradation and photocatalytic CO_2 reduction. *Sol. Energ. Mat. Sol. C.* **2015**, *137*, 175–184.

© 2019 by the authors. Licensee MDPI, Basel, Switzerland. This article is an open access article distributed under the terms and conditions of the Creative Commons Attribution (CC BY) license (http://creativecommons.org/licenses/by/4.0/).
